# Use of Matriderm in 2 cases of Dupuytren’s contracture release

**DOI:** 10.1016/j.jpra.2026.05.028

**Published:** 2026-05-21

**Authors:** Zeljka Roje, Matej Miljak, Filip Luka Mikulic, Boris Zdilar, Zlatko Vlajcic, Rado Zic

**Affiliations:** a Department of Plastic, Reconstructive and Aesthetic Surgery, University Hospital Dubrava, Zagreb, Croatia; b Department of Plastic, Reconstructive and Aesthetic Surgery, Clinical Hospital Sveti Duh, Zagreb, Croatia

**Keywords:** Dupuytren’s contracture, Tenolysis, Matriderm

## Abstract

Dupuytren's contracture is a chronic fibroproliferative disorder of the hand characterized by progressive thickening and shortening of the palmar fascia, leading to flexion deformities of the fingers, particularly the ring and little fingers (1). The exact pathogenesis remains incompletely understood, however, both genetic and environmental factors play significant roles in disease development (2,3). This report highlights the use of Matriderm, a single-layer matrix consisting of collagen and elastin, possibly aiding postoperative outcomes of Dupuytren’s Contracture. A 43-year-old female patient presented with Dupuytren’s contracture of the fifth digit of the left hand. With a positive family history: her father exhibited the most severe form of the condition, possibly exacerbated by his work in the mechanical industry. Matriderm was used in surgeries in both cases, promoting wound healing and adhesiogenic regulation. These properties seemingly helped minimize postoperative scarring, improve joint mobility, and functional outcome.

## Introduction

Dupuytren's contracture (DC) is a chronic fibroproliferative disorder of the hand characterized by progressive thickening and shortening of the palmar fascia, resulting in flexion deformities of the fingers, most commonly the ring and little fingers.^1^ The disease predominantly affects older males of Northern European ancestry and represents one of the most common inherited connective tissue disorders of the hand.^1^ Histopathologically, Dupuytren’s disease is characterized by fibroblast and myofibroblast proliferation, excessive extracellular matrix deposition, and abnormal collagen remodeling within the palmar fascia.^2^

Conservative treatment has traditionally been the primary approach to managing Dupuytren’s contructure, focusing on non-surgical methods: the application of corticosteroid injections, namely, triamcinolone, less so, collagenase injections. Furthermore, with invasive treatements of: needle fasciotomy, and surgical fasciectomy; however, recurrence remains a major therapeutic challenge.^2^

The aim of surgery is to excise or release the pathological fibrous cords within the palmar fascia, thereby improving finger extension and hand function.

With the progression of medical science, there are more medical ingenuities with which it may be possible to develop new treatment options. We present the off-label use of Matriderm—a single-layer dermal matrix composed of collagen and elastin, which mimics the natural extracellular matrix of human skin, promoting and regulating wound healing and tissue regeneration by encouraging endothelial cell migration and capillary formation therefore enhancing angiogenesis and stimulating cellular activity.^7^

## Case report

A 43-year-old woman was evaluated at the Plastic Surgery Outpatient Clinic, Dubrava Clinical Hospital for recurrence of the flexion deformity in the fifth digit of her left hand, following a previous surgical release of the flexor digitorum superficialis (FDS) performed on August 19, 2017. Her medical history revealed a familial predisposition, with her father having undergone surgical correction for flexion contractures due to DC in the third and fourth fingers of the right hand. Additionally, the father's brother had a history of DC, though to a much lesser extent. In this family, the father's case of DC was the most extreme. The father was exposed to vibrational stimuli due to his occupation in the mechanical industry, in contrast to the rest of the family, who were less or not exposed to such occupational environmental factors. The daughter, who worked as a fitness trainer, exhibited a milder form of DC. In contrast, the brother, who held a sedentary job, showed the best outcome, with minimal progression of the condition. This suggests that environmental and occupational factors may affect the clinical course.

**Fig. 1:**
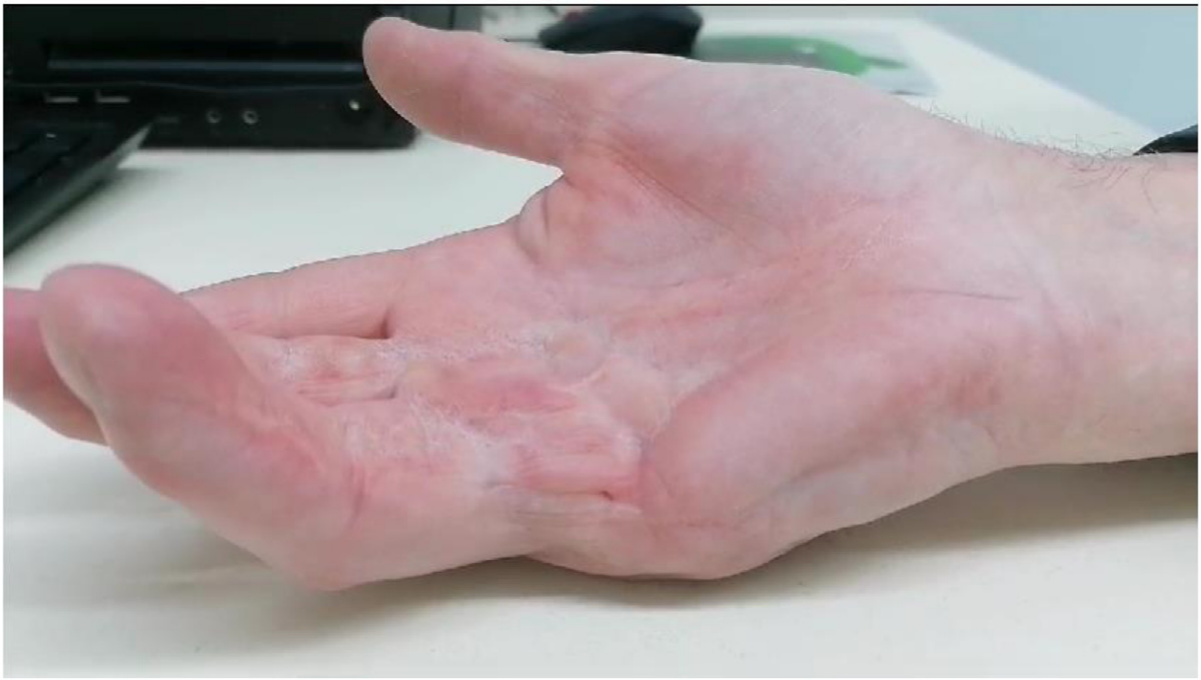
Figure of the patient’s father’s hand several months post-operatively. It depicts the maximally extended digits following surgical treatment with Matriderm peritendinous integration.

Complete release of the contracture was performed on the patient’s father’s hand with preservation of neurovascular bundles. The use of Matriderm during the father's operation was associated with a significantly lower grade of recurrence of postoperative contractures that had been a problem following previous release surgeries and one of the initial reasons for the investigational use of Matriderm in the most recent surgery. The defect was reconstructed using Matriderm, resulting in large improvement in extension (up to the 30°mark) of the affected fingers, digits 3 and 4 (Fig. 1), compared to the outcome of the initial surgery where extension returned to preoperative limits.

The initial procedure done on the patient involved resection of the superficial flexor tendon, removal of the fascia of the fifth finger and stabilization using Kirschner wires. Despite initial success, the deformity recurred, prompting further intervention.

**Fig. 2:**
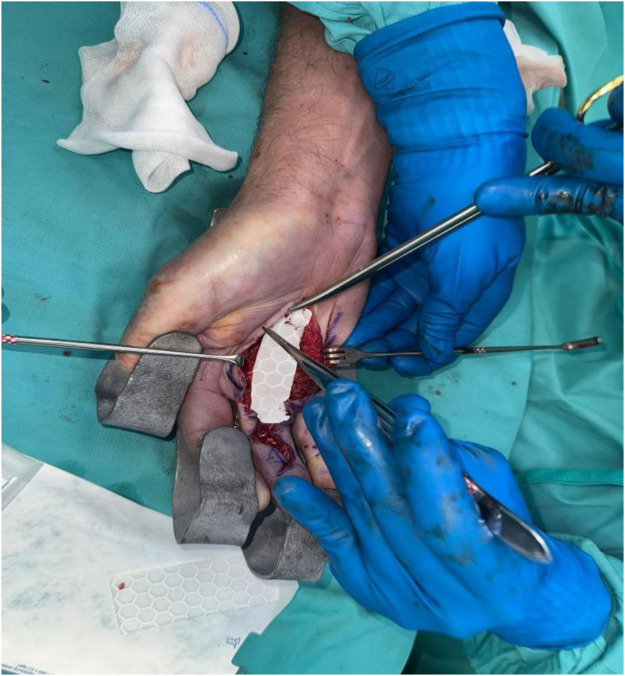
Figure showing the peritendinous placement of Matriderm during the surgical release of Dupuytren’s contracture. All performed surgical release procedures were done using the same protocol.

During the repeat operation 4 years later, a Brunner incision was made on the fifth finger and the palm under a tourniquet. The skin was elevated up to the thickened palmar aponeurosis, which was removed while preserving the neurovascular structures. Then, a tenolysis of the superficial flexor tendon and an arthrolysis of the proximal interphalangeal joint were performed, and the deep flexsor tendon itself was wrapped in Matriderm in a 360° fashion (Fig. 2). In both patients the A2-pulley was partially released at 50 % due to limited gliding of the tendon, which was intraoperatively assessed, as a factor reducing total range of motion. Previous clinical studies indicate that release of the A2-pulley to 50 %, without compromising the pulley’s function. Furthermore, a multiple Z-plasty has been done for the patients during skin closure.

## Discussion

The pathogenesis and treatment of DC remain open to discussion. While some cases occur sporadically, a strong genetic component has been established. Contractures of 30 degrees or more in the metacarpophalangeal joints or any degree of contraction in the proximal interphalangeal joint of any digit have an indication for surgical treatment.

Recent genetic studies have demonstrated a strong hereditary component, with genome-wide association studies identifying multiple susceptibility loci associated with fibrosis-related signaling pathways, particularly the Wnt, Hedgehog, and Notch pathways.^3^^,^^4^ Genes (including *WNT4, SFRP4, RSPO2*, and *MMP14)* have been implicated in disease susceptibility and progression, supporting the concept that dysregulated fibroproliferative signaling contributes to pathological tissue contracture.^3^ Current evidence suggests that genetic factors may account for up to 80 % of disease susceptibility.^5^

Environmental and occupational factors also contribute substantially to disease development. Repetitive manual labor, chronic mechanical loading of the hand, and exposure to hand-transmitted vibration from vibrating tools have been consistently associated with an increased risk of Dupuytren’s disease.^6^ A recent systematic review and meta-analysis demonstrated significantly elevated odds ratios among workers exposed to vibration and heavy manual occupational activities.^6^ Additional recognized risk factors include smoking, alcohol consumption, diabetes mellitus, epilepsy, and advancing age.^5^^,^^7^ Clinically, the disease progresses from painless palmar nodules to fibrous cords and irreversible digital contractures, often resulting in functional impairment and reduced hand dexterity. Current treatment modalities include collagenase injections, needle fasciotomy, and surgical fasciectomy; however, recurrence remains a major therapeutic challenge.^2^

This study is brought upon a novel idea to introduce a tissue barrier (i.e. Matriderm) between the skin and flexor tendons of the hand in zones 2 and 3; to mitigate the need for re-release surgeries that may result during the healing and remodelling process following initial contracture release in this case as a result of DC.

In this case report the father with recurrent stage 2 DC, was a construction worker, exposed to vibrational stimuli for long periods of time. The daughter had a milder presentation, stage 1 DC, despite a physically active, but did not have vibrational exposure. Interestingly, the brother, who had a sedentary lifestyle, showed the least progression, in support of the above hypothesis that environmental factors, especially mechanical stress and vibration, may have considerable influence as deterministic factors in disease severity.

**Fig. 3:**
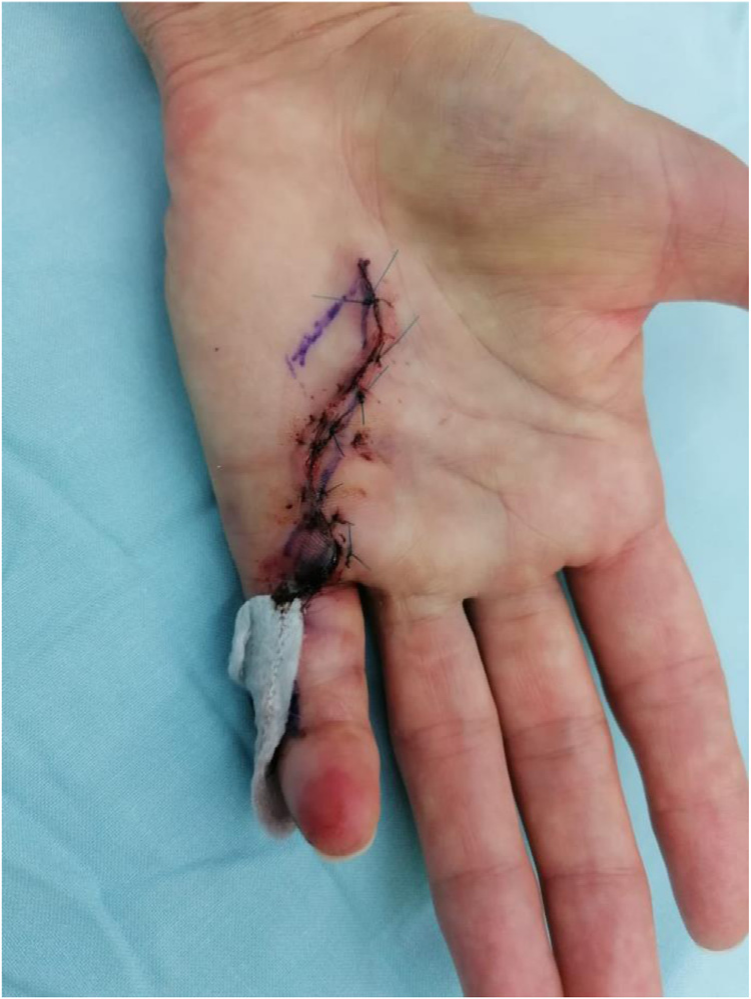
Figure showing the postsurgical suture line in projection of the patient’s 5th digit on the 3rd postoperative day. The figure depicts maximal possible extension of the digits. In addition, no secretions, infection or other complications were present.

**Fig. 4:**
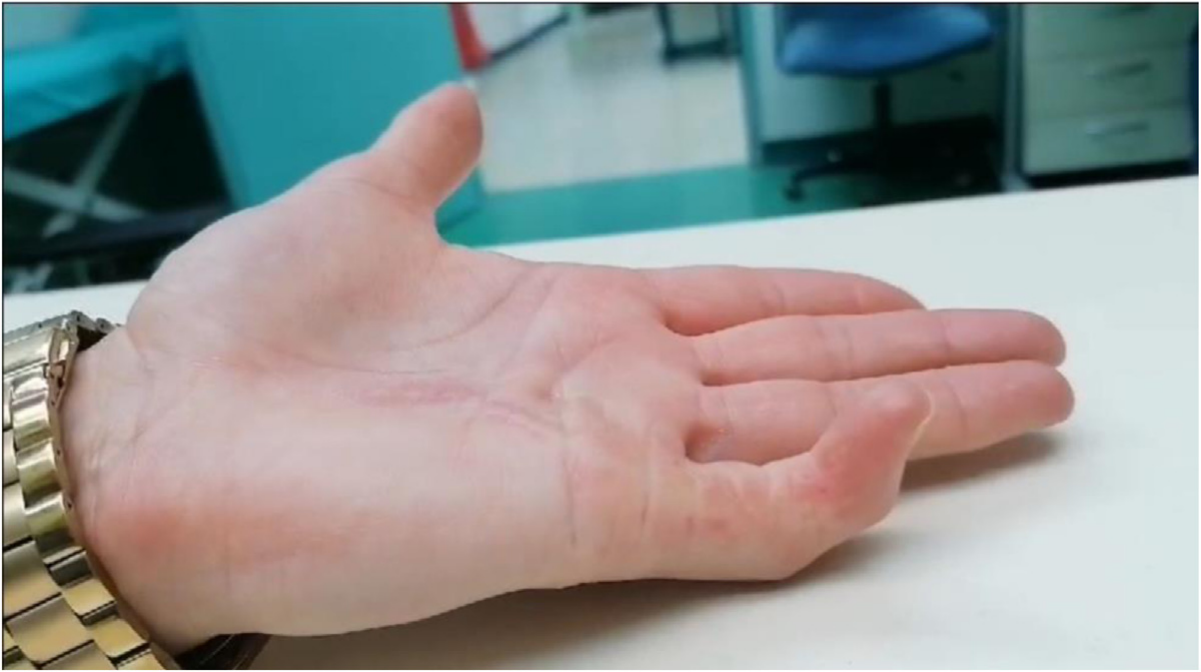
Figure showing a lateral view of the patient’s 5th digit after the first postoperative month. The figure depicts maximal possible extension of the digits. Considerable improvement in range of motion i.e. extension was almost fully recovered in the proximal interphalangeal joint, while in the distal interphalangeal joint up to 50°. In addition, no secretions, infection or other complications were present.

The early postoperative course was without any complications or signs of infection (Fig. 3). At the one-month follow-up, the patient showed satisfactory local wound healing with no neurovascular anomalies, and with considerable improvement in range of motion i.e. extension was almost fully recovered in the proximal interphalangeal joint, while in the distal interphalangeal joint up to 50° (Fig. 4). Flexion of the digits has recovered from 60 % to 90 % in the case of the daughter and from 65 % to 85 % in the case of the father.

This case report suggests that further research should be conducted looking into Matriderm being used as a potential new treatment option for DC and conditions with similar pathogenesis and clinical manifestation. We have proposed a strong argument to conduct a broader clinical trial into Matriderm’s case use mentioned above.

## Funding

None.

## Conflicts of interest

None declared.

## Ethical approval

Not required.
